# Visualization of cardiac uptake of bone marrow mesenchymal stem cell‐derived extracellular vesicles after intramyocardial or intravenous injection in murine myocardial infarction

**DOI:** 10.14814/phy2.15568

**Published:** 2023-03-26

**Authors:** Cynthia M. Xu, Sharif A. Sabe, Rayane Brinck‐Teixeira, Mohamed Sabra, Frank W. Sellke, M. Ruhul Abid

**Affiliations:** ^1^ Cardiovascular Research Center Rhode Island Hospital Providence Rhode Island USA; ^2^ Division of Cardiothoracic Surgery Alpert Medical School of Brown University and Rhode Island Hospital Providence Providence Rhode Island USA

## Abstract

In animal models, human bone marrow mesenchymal stem cell‐derived extracellular vesicles (MSC‐EV) have been found to have beneficial effects in cardiovascular disease, but only when administered via intramyocardial injection. The biodistribution of either intravenous or intramyocardial injection of MSC‐EV in the presence of myocardial injury is uncharacterized at this time. We hypothesized that intramyocardial injection will ensure delivery of MSC‐EV to the ischemic myocardium, while intravenous injection will not. Human bone marrow mesenchymal stem cells were cultured and the MSC‐EV were isolated and characterized. The MSC‐EVs were then labeled with DiD lipid dye. FVB mice with normal cardiac function underwent left coronary artery ligation followed by either peri‐infarct intramyocardial or tail vein injection of 3*10^6^ or 2*10^9^ particles of DiD‐labeled MSC‐EV or a DiD‐saline control. The heart, lungs, liver, spleen and kidneys were harvested 2 h post‐injection and were submitted for fluorescent molecular tomography imaging. Myocardial uptake of MSC‐EV was only visualized after intramyocardial injection of 2*10^9^ MSC‐EV particles (*p* = 0.01) compared to control, and there were no differences in cardiac fluorescence after tail vein injection of MSC‐EV (*p* = 0.5). There was no significantly detectable MSC‐EV uptake in other organs after intramyocardial injection. After tail vein injection of 2*10^9^ particles of MSC‐EV, the liver (*p* = 0.02) and spleen (*p* = 0.04) appeared to have diffuse MSC‐EV uptake compared to controls. Even in the presence of myocardial injury, only intramyocardial but not intravenous administration resulted in detectable levels of MSC‐EV in the ischemic myocardium. This study confirms the role for intramyocardial injection in maximal and effective delivery of MSC‐EV. Our ongoing studies aimed at developing bioengineered MSC‐EV for targeted delivery to the heart may render MSC‐EV clinically applicable for cardiovascular disease.

## Graphical abstract

Intramyocardial delivery appears to be an efficient method of extracellular vesicle (EV) delivery to ischemic myocardium while intravenous injection is not.

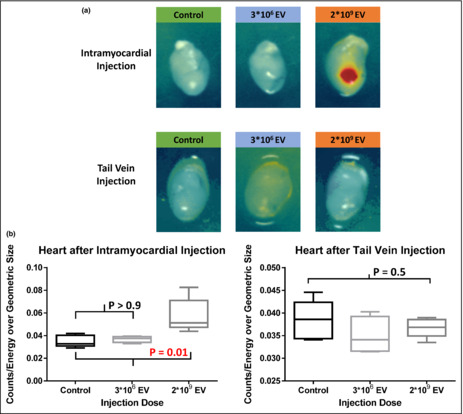

## 1 INTRODUCTION

The role of stem cell‐derived extracellular vesicles (EV) as a therapeutic in cardiovascular disease has been studied for the past decade, and they have been largely found to have beneficial effects on cardiac function in both rodent and large animal models of ischemic disease and heart failure (Guo et al., 2022; La Mantia et al., 2022; Potz et al., 2018; Scrimgeour et al., 2019; Spannbauer et al., 2020; Xiao et al., 2022). Under ischemic conditions such as in myocardial infarction, EVs can prevent delayed injury, promote angiogenesis and aid in tissue remodeling and function through various mechanisms (Zheng et al., 2021). However, a significant barrier to the use of EVs in a clinical setting is the method of delivery. As of now, the most effective and reliable mode of delivery is via intramyocardial injection, which would require thoracotomy in a patient population that may not be able to tolerate or achieve net benefit from an operation. Less invasive methods of delivery, such as intravenous, may not confer meaningful benefits in cardiovascular disease (Chen et al., 2021; Scrimgeour et al., 2020). However, it is not known if there are any significant differences in EV biodistribution depending on the route of administration in animal models of cardiovascular disease. This is the first study to attempt to compare the biodistribution of human bone marrow mesenchymal stem cell‐derived extracellular vesicles (MSC‐EV) after intramyocardial versus intravenous injection in an animal model of myocardial ischemia.

EVs are secreted by almost all cell types, and contain many bioactive molecules, including proteins and nucleic acids. EVs appear to be taken up by cells through endocytic routes and have key roles in cell‐to‐cell communication which affect recipient cells by influencing gene expression, signaling pathways, and cellular phenotype/behavior (Fu et al., 2020; Mulcahy et al., 2014). MSC‐EVs, as well as EVs derived from other progenitor sources, have regenerative and immunomodulatory properties, and been delivered via intramyocardial, intravenous, intracoronary, and intrapericardial routes (Dabrowska et al., 2021; Spannbauer et al., 2020). Intravenous and intracoronary injections have had mixed results; intrapericardial is not as well studied (Gallet et al., 2017; López et al., 2020; Scrimgeour et al., 2020; Spannbauer et al., 2020; Wang et al., 2021; Zhu et al., 2021). Additionally, when EVs are injected intravenously (either tail vein or retro‐orbital), they have a circulation half‐life well within 60 minutes, and are readily taken up by macrophages, which congregate in the liver or spleen (Kooijmans et al., 2016; Mentkowski & Lang, 2019; Parada et al., 2021; Wen et al., 2019). Other factors to consider in predicting the location of EV uptake are the origin of the progenitor cell from which the EVs were isolated, and the presence of cellular damage as injured cells more readily take up EVs than healthy cells (Stik et al., 2017; Wen et al., 2019).

Previous biodistribution experiments have investigated the uptake of EVs in mouse models and detected no cardiac uptake with intravenous injection—these models included MSC‐EVs delivered via tail vein injection in a murine radiation model and cardiosphere‐derived EVs delivered via retro‐orbital injection in wild‐type non‐infarcted mice (Mentkowski & Lang, 2019; Wen et al., 2019). Only with intramyocardial injection were cardiosphere‐derived EVs localized to the heart in the non‐infarcted mice (Mentkowski & Lang, 2019). No cardiac injury was present in these models. Therefore, the aim of this study is to investigate whether the routes of administration, intramyocardial versus intravenous, affect MSC‐EV uptake in the presence of myocardial injury.

## 2 METHODS

### 2.1 Human bone marrow mesenchymal stem cell (HBMSC) culture

HBMSC were purchased from Lonza (PT‐2501), grown in T175 cm^2^ flasks to passage 6 with 30 ml Mesenchymal Stem Cell Growth Medium BulletKit (MSCGM) (Lonza, PT‐3001), and cultured per the manufacturer's instructions. At passage 7 (consistent with previously used protocols from our group), the cells were split into 100‐mm dishes and cultured with 10 ml of MSCGM. The cells were placed in a humidified incubator at 37°C with 5% CO_2_.

### 2.2 MSC‐EV isolation

At passage 7 and 80%–90% confluency (approximately 6.5–7.2 million cells), the MSCGM was removed and was replaced with 7 ml fresh MSCGM. The cells were then placed in an airtight humidified hypoxia chamber (Billups‐Rothenberg, MIC‐101) containing 95% N_2_ and 5% CO_2_. Hypoxia was induced by connecting the chamber's inflow cannula to a gas tank containing 95% N_2_ and 5% CO_2_ with a flow rate of 20 L/min with the outflow cannula open for 7 min to wash out O_2_. After 7 min, the outflow cannula was clamped shut first, then the inflow cannula was clamped and the gas flow was turned off. The chamber was then placed at 37°C for 24 h. Afterwards, the hypoxia chambers were opened and the media was collected, which was then centrifuged at 2000× *g* to remove the cell debris. The media then underwent ultracentrifugation (WX Ultra Centrifuge with Sorvall AH‐629 rotor) at 100,000× *g* for 70 min to isolate the MSC‐EV pellet. The MSC‐EV were then washed with Dulbecco's Phosphate Buffered Saline (PBS) and centrifuged at 100,000× *g* for another 70 min. The MSC‐EV were re‐suspended in PBS with 1% dimethylsulfoxide, and stored at −80°C (Potz et al., 2018; Wen et al., 2019).

### 2.3 MSC‐EV characterization studies

The MSC‐EV were evaluated by electron microscopy (FEI Morgagni 268) after fixation in 2% paraformaldehyde for 20 min. The MSC‐EV were washed with PBS, fixed with 1% glutaraldehyde and contrasted in 4% uranyl acetate. With the NanoSight NS500 (Malvern Instruments), the size, number and distribution of the MSC‐EVs were determined. The following MSC‐EV markers were evaluated via western blot: CD81 (Cell Signaling, 52892S), CD9 (Cell Signaling, 13403S), Alix (Cell Signaling, 92880S), GAPDH (Cell Signaling, 97166S), heat shock protein 70 (HSP70) (Cell Signaling, 4872T), and albumin (Cell Signaling, 4929S).

### 2.4 Fluorescent labeling of MSC‐EV

Fluorescent‐labeled MSC‐EVs as well as a negative control were prepared. The MSC‐EV were thawed on ice. PBS was added to the MSC‐EV to make a total volume of 1 ml, and to this 5 ul of Vybrant DiD Cell‐Labeling Solution (Invitrogen, V22887) was added (Wen et al., 2019). For the negative control (DiD‐saline), 5 ul of the labeling solution was added to 1 ml of PBS. Light exposure was minimized during this entire process. These solutions were incubated at 37°C for 30 min. The solutions were then transferred to 2 separate ultracentrifuge tubes with an additional 30 ml PBS each. The solutions were washed two times with 30 ml PBS and underwent two ultracentrifuge cycles at 100,000× *g* for 1 h each. After the final wash, the DiD‐labeled MSC‐EV and negative control were re‐suspended with PBS, and aliquoted for intramyocardial or tail vein injection.

### 2.5 Animals

Female and male FVB/NCrl mice (6–8 weeks old) from Charles River (Stock No. 207) were used in this study, with an *n* = 5 per experimental group. The animals were housed at the Coro Building Barrier facility, acclimatized appropriately and fed a normal diet. All experimental procedures carried out in accordance with the protocol approved by Institutional Animal Care and Use Committee (Protocol 1844667/CMTT# 5017‐22). The experiments were carried out over several weeks by a single experienced surgeon with freshly prepared fluorescent‐labeled MSC‐EVs or DiD‐saline.

### 2.6 Echocardiogram

The mice underwent pre‐operative echocardiogram (Vevo 2100, FUJIFILM VisualSonic Inc.). Under 2% isoflurane anesthesia, normothermia, and heart rate maintenance between 400–600 beats per minute, left heart systolic function was evaluated via two‐dimensional parasternal long axis views with left ventricular trace measurements to determine the left ventricular ejection fraction (LVEF).

### 2.7 Surgical procedure: Left anterior descending coronary artery (LAD) ligation and injection

Anesthesia was induced in the mice with 3% isoflurane and ketamine (100 mg/kg), and the mice were intubated and ventilated (MiniVent Type 845, Harvard Apparatus). Buprenorphine SR (1 mg/kg) was administered subcutaneously in the dorsal fat pad. Isoflurane was then maintained at 2%. The heart was exposed with a left thoracotomy, and the LAD was ligated with an 8–0 nylon suture 2–3 mm below the left atrial appendage (Reichert et al., 2017). Successful ligation was confirmed with subsequent blanching and dyskinesia.

The mice were then allocated to one of six groups – (1) intramyocardial injection with DiD‐saline (*n* = 5), (2) intramyocardial injection with 3*10^6^ particles DiD‐labeled MSC‐EV (*n* = 4), (3) intramyocardial injection with 2*10^9^ particles DiD‐labeled MSC‐EV (*n* = 5), (4) tail vein injection with DiD‐saline (*n* = 5), (5) tail vein injection with 3*10^6^ particles DiD‐labeled MSC‐EV (*n* = 4), and (6) tail vein injection with 2*10^9^ particles DiD‐labeled MSC‐EV (*n* = 5). The MSC‐EV particles were quantified by nanoparticle‐tracking analysis.

Immediately after LAD ligation, the injection was performed prior to closing the thoracotomy. To perform the intramyocardial injection, a Neuros Syringe (Hamilton, 1183U32) was used to inject 5 μl of DiD‐labeled MSC‐EV or DiD‐saline into the peri‐infarct area. To perform the tail vein injection, a 0.5 ml insulin syringe was used to inject 200 μl of DiD‐labeled MSC‐EV or DiD‐saline.

The thoracotomy was then closed with 6–0 vicryl suture and the pneumothorax was evacuated. The skin was closed with absorbable sutures. The mice were successfully weaned from anesthesia and extubated.

### 2.8 Organ harvest and fluorescent molecular tomography (FMT) imaging

Two hours post‐injection, the mice were euthanized via carbon dioxide inhalation and cervical dislocation. The heart, lungs, liver, kidneys and spleen were then dissected and transferred into separate Eppendorf tubes with cold PBS. The organs were imaged with the FMT 4000 imaging system (PerkinElmer) to obtain fluorescence reflectance images. The fluorescence was quantified with TrueQuant v3.0 (PerkinElmer) by measuring the counts/energy, normalized by the geometric size captured of the organ.

### 2.9 Statistical analysis

For the statistical analysis of the LVEF obtained via echocardiography, the Shapiro–Wilk and Kruskal–Wallis *H* tests were performed. For the analysis of the organ fluorescence quantification, the Shapiro–Wilk test, Kruskal–Wallis *H* test and post hoc Dunn's Multiple Comparison test were used.

## 3 RESULTS

### 3.1 MSC‐EV characterization

**FIGURE 1:**
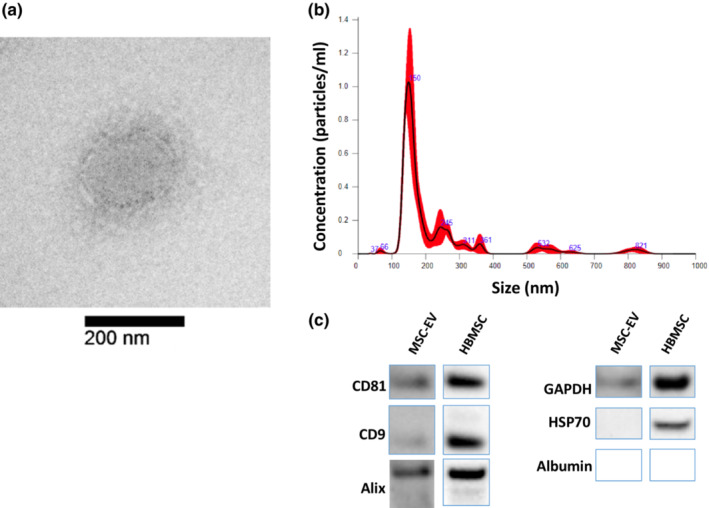
Human bone marrow mesenchymal extracellular vesicle (MSC‐EV) characterizations. (a). Electron microscopy image of MSC‐EV (scale bar = 200 nm; magnification 54,800x). (b). MSC‐EV fractions determined by nanoparticle‐tracking analysis, demonstrating mean particle size to be 217.3 nm ± 10.9 nm. c. Western blot images of CD81, CD9, Alix, GAPDH, HSP70, and albumin of MSC‐EV lysates and human bone mesenchymal stem cell (HBMSC) lysates serve as qualitative measures of protein marker presence. CD81, CD9, Alix, and GAPDH were identified in the MSC‐EV. HSP70, a promiscuous cytosolic protein, and albumin, a marker of contamination, were not identified in the MSC‐EV lysates. The absence of albumin confirmed the purity of the isolated MSC‐EVs. MSC‐EV and HBMSC bands are shown in separate images given the need for vastly different exposure times during imaging. See Supplemental Figures for full western blot images.

The MSC‐EVs were visualized via electron microscopy (Figure 1a), and their size and concentrations were quantified via nanoparticle‐tracking analysis (mean EV size was 217.3 nm ± 10.9 nm; Figure 1b). Western blot analysis confirmed the presence of the following MSC‐EV markers: the transmembrane proteins CD81 and CD9 (Figure 1c); and the cytosolic proteins Alix and GAPDH (Théry et al., 2018; Wen et al., 2019). Neither HSP70, which has promiscuous incorporation in cytosolic protein content, nor albumin, a marker of contamination, were identified in the MSC‐EV lysates. The lack of albumin confirmed purity of the isolated MSC‐EVs.

### 3.2 Normal pre‐operative systolic function was confirmed by echocardiogram

Normal left heart systolic function was confirmed via echocardiography, with LVEF identified within normal parameters. There were no significant differences in LVEF between the three MSC‐EV dosage animal groups (negative control, 3*10^6^ MSC‐EV, 2*10^9^ MSC‐EV) (*p* = 0.9). The mean LVEF for the three dosage groups (negative control, 3*10^6^ MSC‐EV, 2*10^9^ MSC‐EV) were 67% ± 4.5%, 68% ± 3.3%, and 67% ± 3.2%, respectively.

### 3.3 Organ fluorescence detection showed cardiac uptake of MSC‐EV only after intramyocardial injection

**FIGURE 2:**
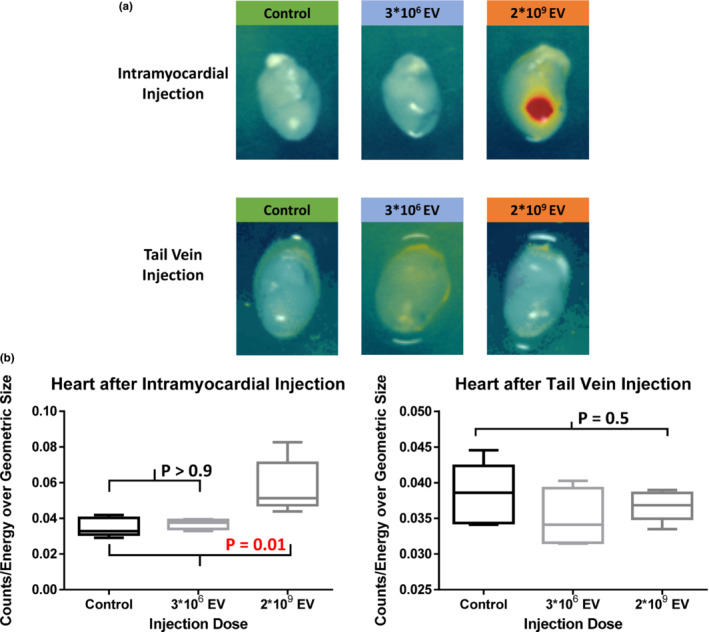
Fluorescence molecular tomography of the heart after intramyocardial injection or tail vein injection of either a negative DiD‐saline control or DiD‐labeled human bone mesenchymal stem cell‐derived extracellular vesicles (MSC‐EV). (a) Uptake of MSC‐EV was only detected after intramyocardial (upper panels) injection of 2*10^9^ particles. No fluorescence was detected in the heart after tail vein (lower panels) injection of either 3*10^6^ or 2*10^9^ particles MSC‐EV. (b) Significantly higher fluorescence was detected only after intramyocardial injection of 2*10^9^ DiD‐labeled MSC‐EV compared to control (*p* = 0.01). No significant fluorescence uptake was seen after tail vein injection of either low (3*10^6^ particles) or high dose (2*10^9^ particles) of MSC‐EV (*p* = 0.5). Thus, intramyocardial injection more reliably delivers MSC‐EV to the ischemic heart. The Shapiro–Wilk test, Kruskal–Wallis *H* test and post hoc Dunn's multiple comparisons test were used for statistical analysis.

**FIGURE 3:**
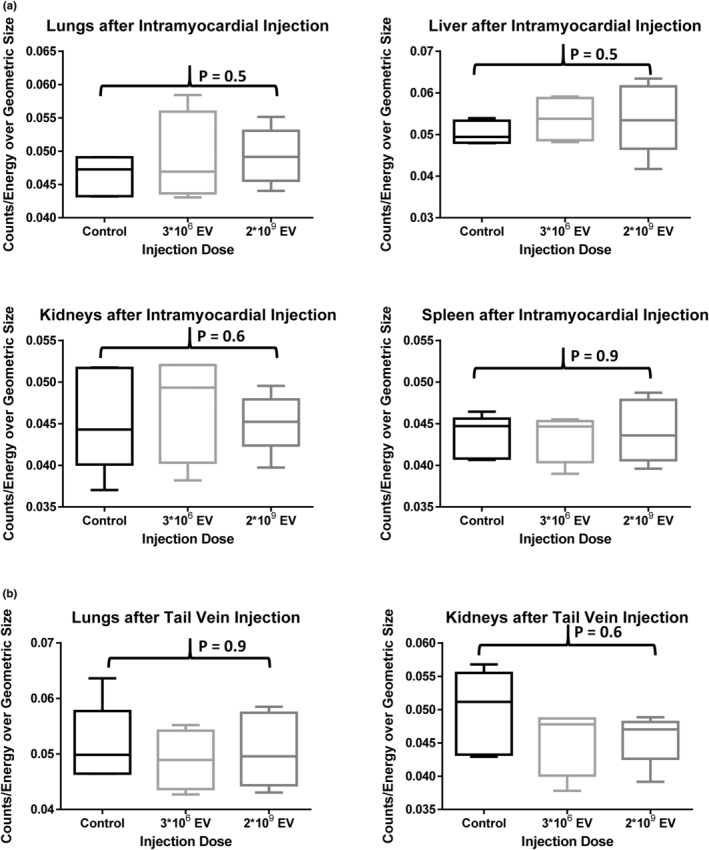
Organs with no detectable immunofluorescence after (a) intramyocardial injection of DiD‐labeled human bone marrow mesenchymal stem cell‐derived extracellular vesicles (MSC‐EV) and (b) tail vein injection of MSC‐EV (see Figure 2 for heart). After intramyocardial injection, no MSC‐EV uptake was visualized in the lungs, liver, kidneys or spleen (*p* = 0.5, *p* = 0.5, *p* – 0.9 and *p* = 0.6, respectively). After tail vein injection, no MSC‐EV uptake was visualized in the heart (see Figure 2), lungs or kidneys (*p* = 0.9, *p* = 0.6, respectively). Thus, intramyocardial injection did not result in systemic delivery of MSC‐EV at 2 h, and the MSC‐EVs were not detected in the heart, lungs or kidneys 2 h after intravenous injection. The Shapiro–Wilk test and Kruskal–Wallis *H* test were used for analysis.

In the intramyocardial injection groups, myocardial MSC‐EV uptake 2 h post‐injection was seen only with the injection of 2*10^9^ particles, with uptake significantly increased from control (*p* = 0.01). Intramyocardial injection of 3*10^6^ particles of DiD‐labeled MSC‐EV did not demonstrate fluorescent uptake compared to control (*p* > 0.9; Figure 2). No significant fluorescence was detected in any other organs (lungs, liver, kidneys and spleen) 2 h post‐intramyocardial injection using 3*10^6^ or 2*10^9^ particles (*p* = 0.5, *p* = 0.5, *p* = 0.6, *p* = 0.9, respectively; Figure 3a).

**FIGURE 4:**
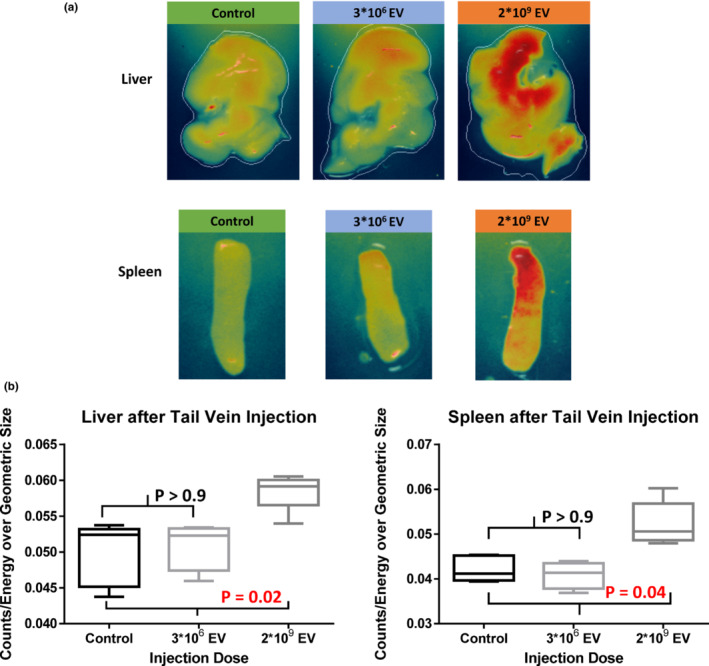
Immunofluorescence detected in the liver and spleen after tail vein injection of human bone marrow mesenchymal stem cell‐derived extracellular vesicles (MSC‐EV). (a) Representative images of the liver (upper panels) and spleen (lower panels) depicting increased organ fluorescence after the injection of 2*10^9^ particles of DiD‐labeled MSC‐EV but not with 3*10^6^ particles of MSC‐EV. (b) Quantification of immunofluorescence in the liver and spleen, which showed significantly increased levels of fluorescence after the MSC‐EV injection of 2*10^9^ particles, compared to control (*p* = 0.02, *p* = 0.04, respectively). Thus, intravenous injection of MSC‐EV results in delivery to the liver and spleen. The Shapiro–Wilk test, Kruskal–Wallis *H* test and post hoc Dunn's multiple comparisons test were used for statistical analysis.

Following tail vein injection of MSC‐EV, no myocardial uptake was seen (*p* = 0.5; Figure 2). As expected, there was increased fluorescence detected in the liver (*p* = 0.02) and spleen (*p* = 0.04) after injection of 2*10^9^ particles MSC‐EV compared to control. No increased fluorescence was detected in the liver or spleen after the tail vein injection of 3*10^6^ particles MSC‐EV (*p* > 0.9, *p* > 0.9, respectively; Figure 4). No significant fluorescence was detected in the lungs or kidneys after tail vein injection of MSC‐EVs using 3*10^6^ or 2*10^9^ particles (*p* = 0.9, *p* = 0.6, respectively; Figure 3b).

## 4 DISCUSSION

To the best of our knowledge, prior to this study, the biodistribution of MSC‐EVs after intramyocardial versus intravenous injection in a myocardial ischemia model had not been investigated before. Here we demonstrated that intramyocardial injection most effectively delivered the MSC‐EV dose to the ischemic myocardium, and that tail vein injection did not result in detectable levels of MSC‐EV in the heart despite the presence of myocardial injury.

Previous studies have shown that cellular injury can increase the uptake of EVs. For example, in murine models of glycerol‐induced acute kidney injury and radiation injury, MSC‐EVs had detectable heightened accumulation in the kidneys and hematopoietic organs, respectively, after tail vein injection (Grange et al., 2014; Wen et al., 2019). Unfortunately, our model does not demonstrate increased uptake in the heart after acute myocardial ischemia and immediate intravenous injection. Numerous studies have shown optimal EV therapeutic benefits in ischemic cardiovascular disease consistently with only intramyocardial injection but not intravenous injection (though this route may have some effects with very high dosing) (Chen et al., 2021; Gallet et al., 2017; Potz et al., 2018; Scrimgeour et al., 2020; Vandergriff et al., 2015). One explanation could be that in the presence of ischemia, EVs administered systemically are not able to reach their destination due to impaired blood flow. Additionally, the coronary capillaries, unlike the sinusoidal hepatic and splenic capillaries, have tight junctions that may limit EV uptake by the myocardium. Another factor to consider is the timing of the MSC‐EV administration – it is unclear when the cardiomyocytes and cardiac endothelial cells release injury signals that could potentially attract the EVs with subsequent increased uptake into the myocardium. Future studies could explore different time points of EV injection, or ischemia/reperfusion models with resulting increased endothelial permeability.

As expected, hepatic and splenic uptake was detected after tail vein injection of MSC‐EV – this finding is consistent with many previous experiments that demonstrated increased fluorescence of the liver and spleen after intravenous injection, which is due to the macrophage uptake of the EVs and subsequent macrophage accumulation in the liver and spleen. Lung uptake was not detected 2 h after injection, but previous studies show that most of the lung signal rapidly decreases after 1 h (Kang et al., 2021; Wen et al., 2019).

Limitations of this study include the lack of evaluation of the consequences of intramyocardial versus tail vein MSC‐EV administration in this myocardial ischemia model beyond 2 h—future experiments could examine post‐operative cardiac function and the effects on infarct size and angiogenesis. Also, the EV dosages administered in fluorescence uptake studies are markedly supra‐therapeutic given the limits of the detection devices—thus the lack of immunofluorescence may not equate to the absence of EVs. However, we can still conclude that intramyocardial injection results in the maximal dose delivered to the heart.

In conclusion, intramyocardial injection appears to be the optimal mode of delivery of MSC‐EV to ischemic myocardium. This study will help future experiments treat myocardial ischemia with MSC‐EVs by optimization of the MSC‐EV route of administration, and the murine model used in this study can be developed to be a high throughput vehicle for testing the efficacy of various kinds of MSC‐EVs, as well as testing EVs bioengineered to increase cardiac uptake.

## AUTHOR CONTRIBUTIONS

C.X. conceived the idea along with experimental design, conducted the majority of the experiments (cell culture, extracellular vesicle isolation and labeling, extracellular vesicle characterization studies, echocardiogram, surgeries, fluorescent imaging and organ harvest), performed the data analysis, and drafted the manuscript. S.S. helped perform experiments (cell culture, extracellular vesicle isolation and fluorescent imaging) and edited the manuscript. R.B.T. helped with the experiment conception, animal experiments (surgeries), and edited the manuscript. M.S. helped perform experiments (extracellular vesicle isolation and organ harvest) and reviewed the manuscript. F.W.S. contributed to the conception of the experiment and reviewed the manuscript. M.R.A. contributed to the conception of the experiment, supervised the project including experimental planning and data collection/analysis, reviewed the manuscript and provided critical feedback.

## FUNDING INFORMATION

Funding for this research was provided by the National Heart, Lung, and Blood Institute (NHLBI) 1R01HL133624 and 2R56HL133624‐05 (M.R.A.); R01HL46716 and R01HL128831‐01A1 (F.W.S.); T32 GM065085‐10 (J.A.).

## DISCLOSURES

None.

## ETHICS STATEMENT

This study was approved by the Institutional Animal Use and Care Committee at Rhode Island Hospital (ref. no. 501722/2022).

## Supporting information


Figure S1.
