# Supplementary material for: Visualization of cardiac uptake of bone marrow mesenchymal stem cell‐derived extracellular vesicles after intramyocardial or intravenous injection in murine myocardial infarction
Source: Physiol Rep. 2023 Mar 26;11(6):e15568. doi: 10.14814/phy2.15568 (PMC10040402; doi:10.14814/phy2.15568)
Supplement: Supplementary file 1 — Figure S1. [file PHY2-11-e15568-s001.pptx]

## Slide 1
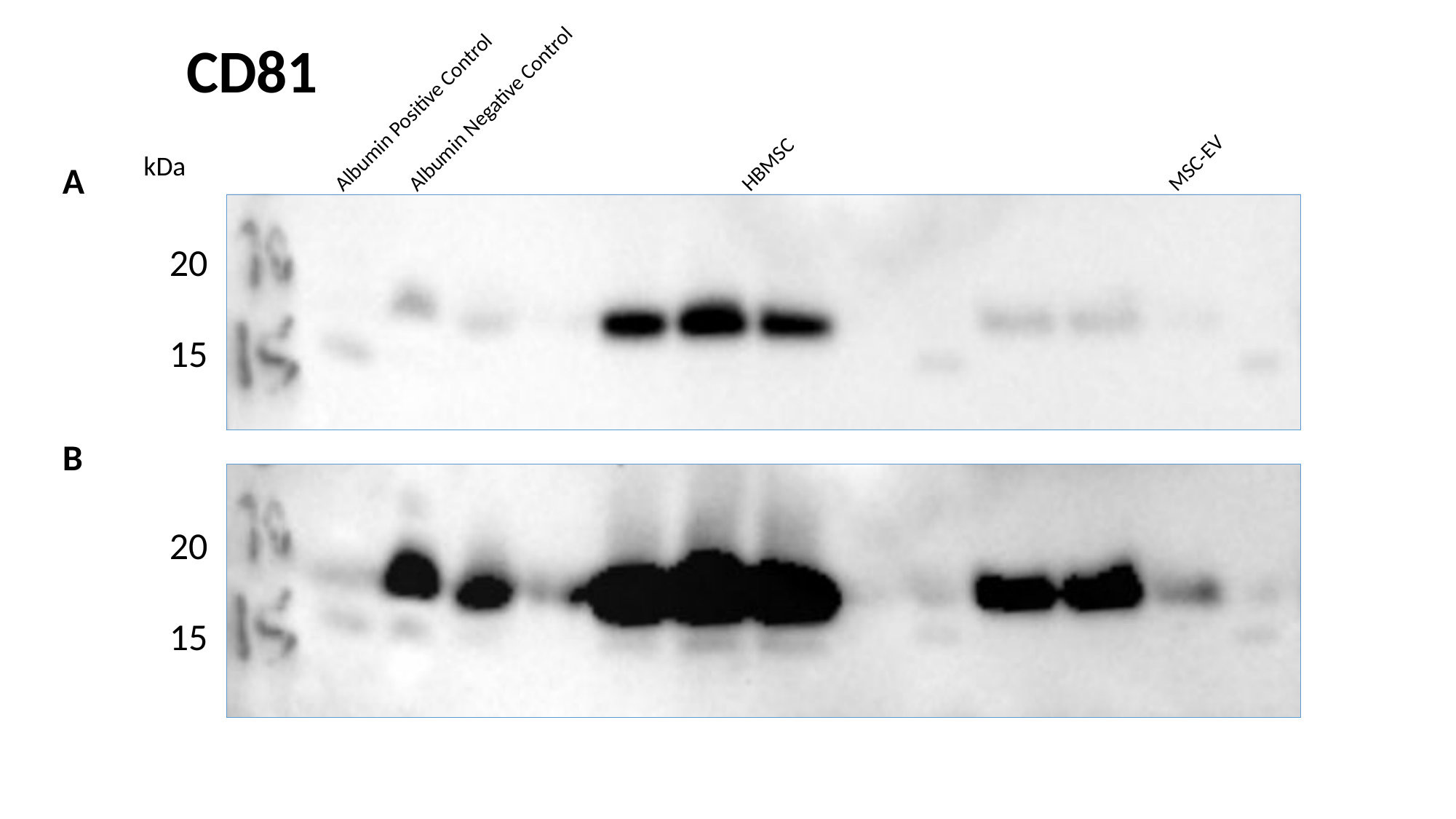

CD81
HBMSC
Albumin Negative Control
MSC-EV
Albumin Positive Control
kDa
A
20
15
B
20
15

## Slide 2
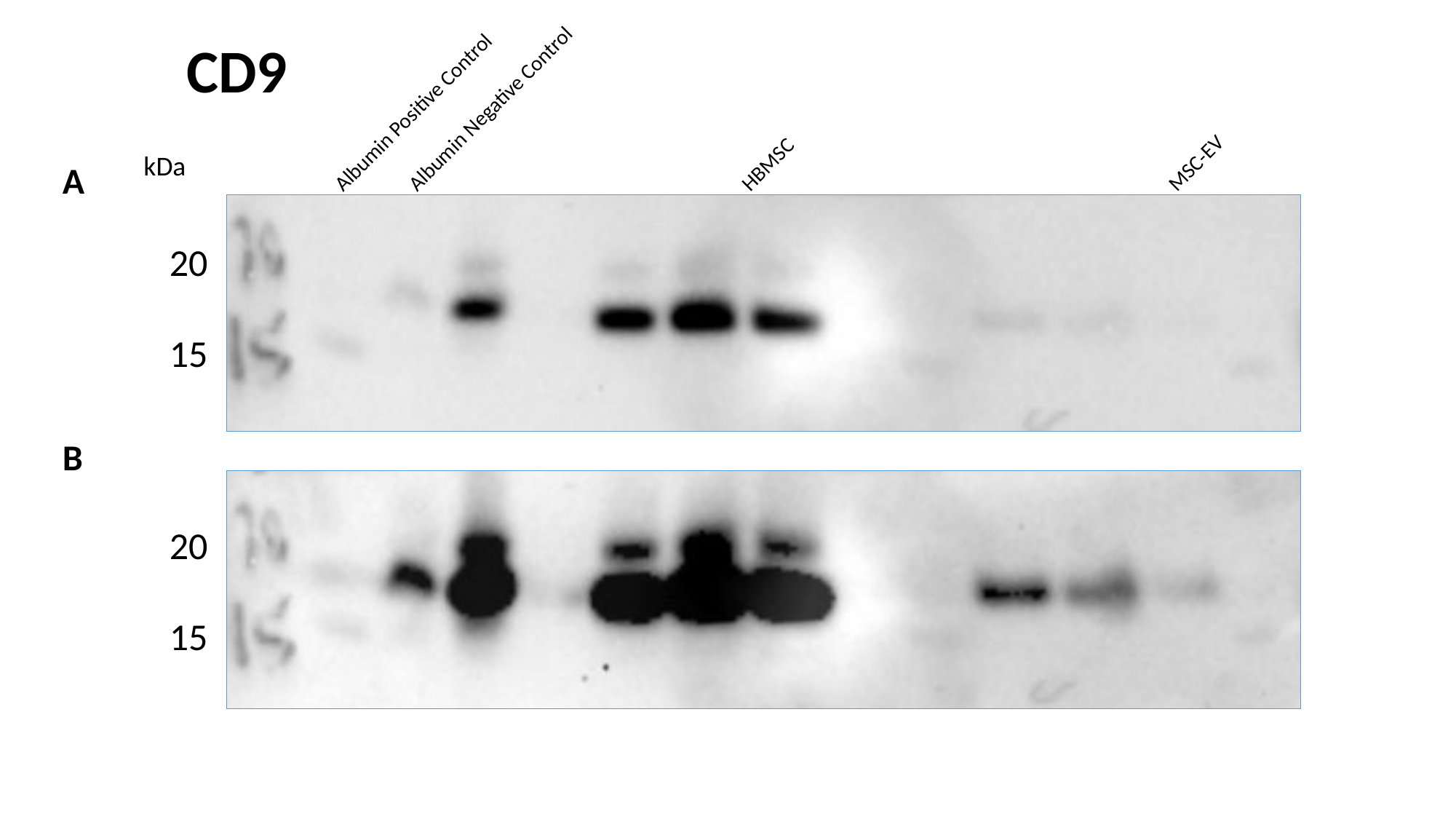

CD9
HBMSC
Albumin Negative Control
MSC-EV
Albumin Positive Control
kDa
A
20
15
B
20
15

## Slide 3
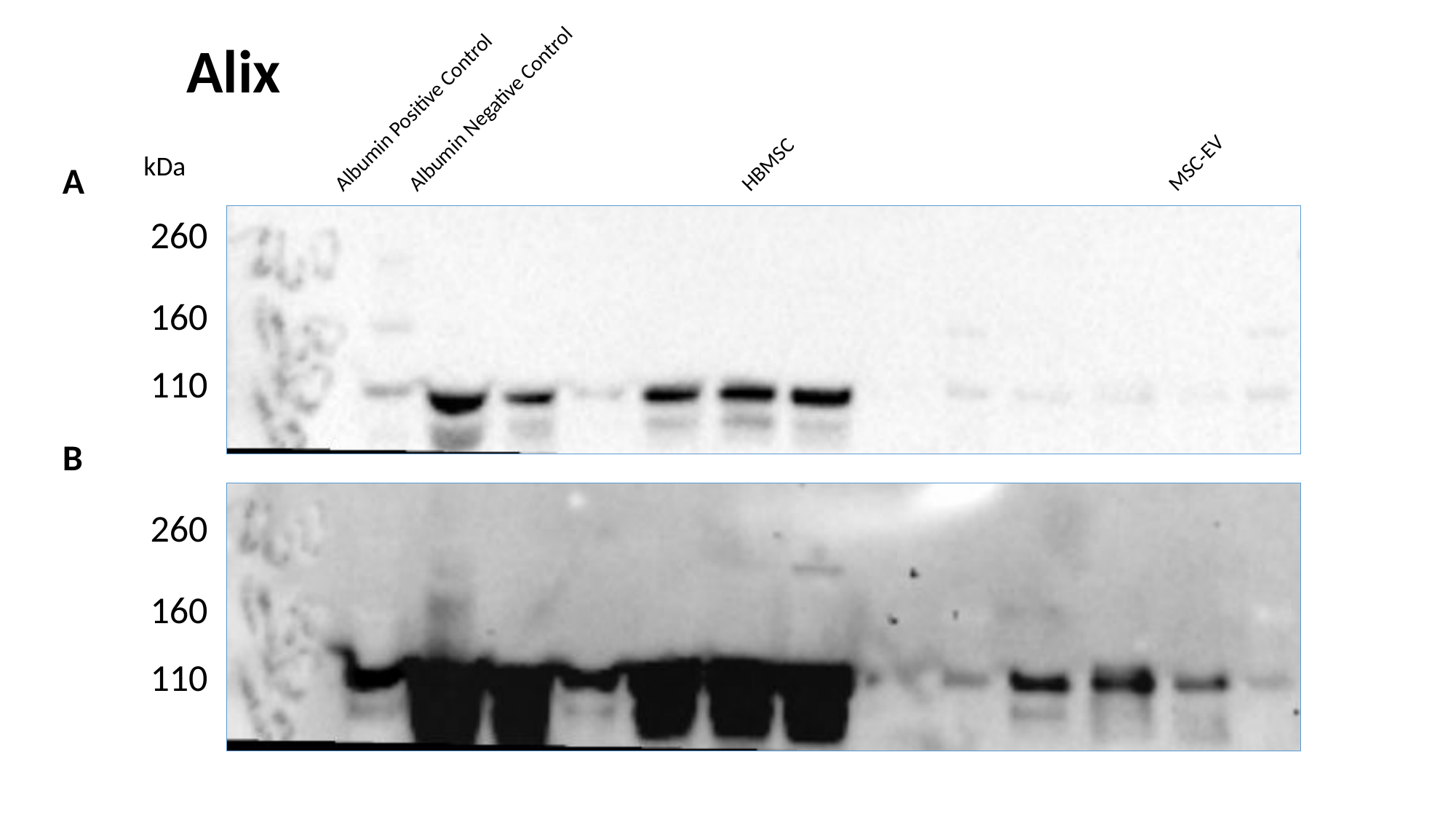

Alix
HBMSC
Albumin Negative Control
MSC-EV
Albumin Positive Control
kDa
A
260
160
110
B
260
160
110

## Slide 4
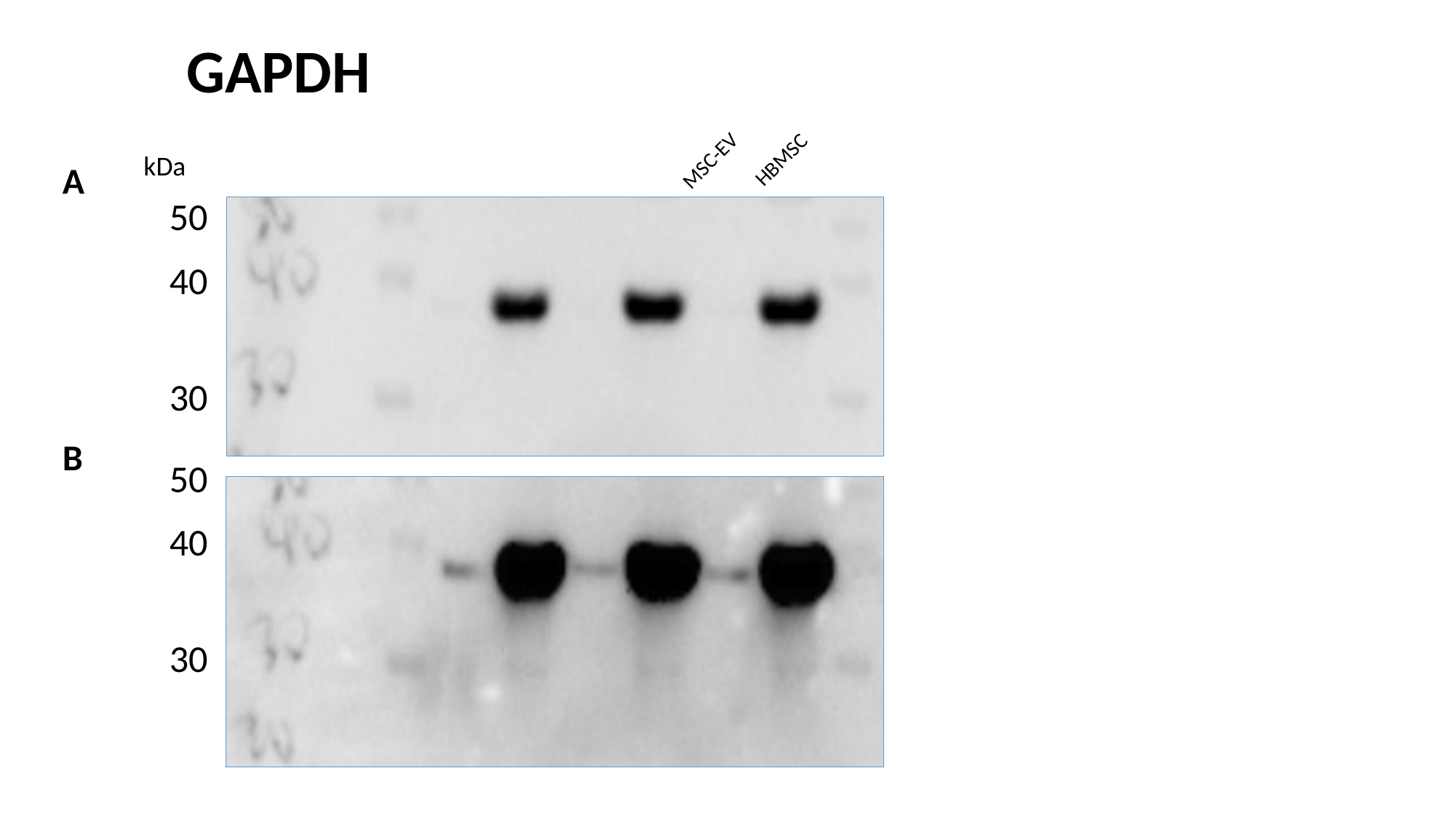

GAPDH
HBMSC
MSC-EV
kDa
A
50
40
30
B
50
40
30

## Slide 5
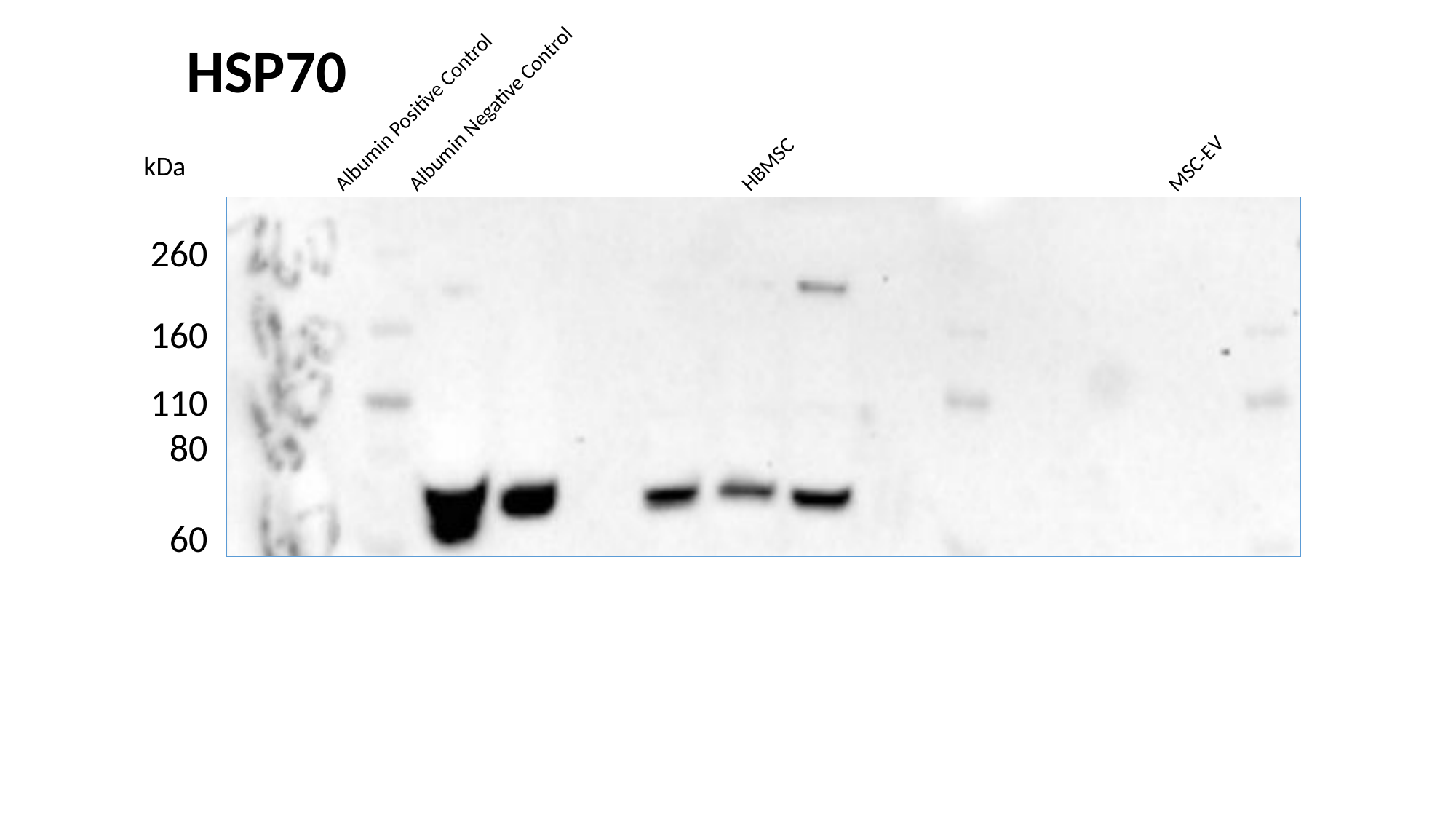

HSP70
HBMSC
Albumin Negative Control
MSC-EV
Albumin Positive Control
kDa
260
160
110
80
60

## Slide 6
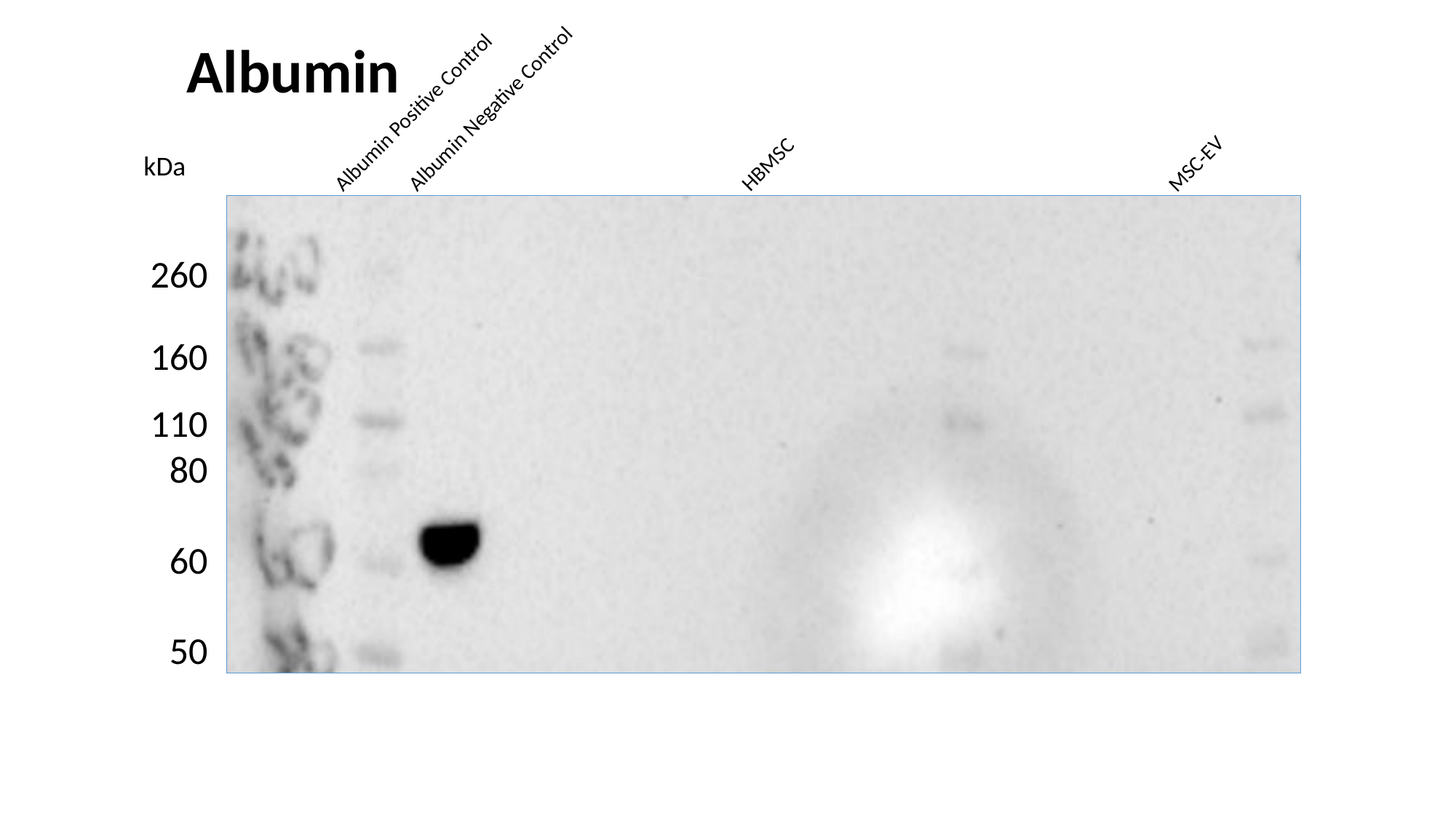

Albumin
HBMSC
Albumin Negative Control
MSC-EV
Albumin Positive Control
kDa
260
160
110
80
60
50
